# A single-cell RNA-seq dataset characterizing cellular diversity in healthy equine skin

**DOI:** 10.1038/s41597-026-08404-8

**Published:** 2026-09-30

**Authors:** Srinivas Akula, Birong Zhang, Miia Riihimäki, Sara Wernersson, Amanda Raine

**Affiliations:** 1 Department of Animal Biosciences, Swedish University of Agricultural Sciences, Uppsala, Sweden; 2 Department of Medical Sciences, Uppsala University, Uppsala, Sweden, Science for Life Laboratory, Uppsala University, Uppsala, Sweden; 3 Department of Clinical Sciences, Swedish University of Agricultural Sciences, Uppsala, Sweden

## Abstract

The skin serves as the primary barrier tissue in horses and is frequently affected by immune-mediated dermatological conditions, most notably insect bite hypersensitivity (IBH). Yet, the cellular composition and transcriptional landscape of normal equine skin have never been characterized at single-cell resolution. Here, we present a single-cell RNA sequencing dataset of healthy equine skin comprising 85,574 high-quality transcriptomes from two horses, with one skin biopsy collected from each horse, divided into two portions for independent processing via manual or automated tissue dissociation. The dataset resolved 22 transcriptionally distinct cell populations, encompassing keratinocyte subpopulations that reflect discrete epidermal differentiation states, adnexal epithelial lineages, stromal and vascular compartments, and resident immune cell types. Cell-type identities are supported by marker gene expression, KEGG pathway enrichment analysis, and functional module scoring. This dataset constitutes the first single-cell transcriptomic reference of normal equine skin, enabling investigations into equine dermatological diseases, wound healing, immune responses and comparative skin biology.

## Background & Summary

The skin is the largest organ of the equine body. It serves multiple critical functions, including providing a protective barrier against environmental insults, regulating body temperature, mediating tactile sensation, and supporting immunity^1,2^. Epidermis, the outer layer of the skin, is a keratinised stratified squamous epithelium predominantly composed of keratinocytes, along with melanocytes, Langerhans cells, and Merkel cells. The underlying dermis is a thicker layer of connective tissue that contains hair follicles, various glands, and sensory nerve receptors. The cell population in the dermal connective tissue is dominated by fibroblasts, which synthesise the extracellular matrix. Based on histological examinations, healthy equine dermis also contains immune cells, predominantly in the perivascular space, including mast cells, macrophages, dendritic cells, and lymphocytes^1–4^.

Horses are affected by a range of dermatological conditions, among which insect bite hypersensitivity (IBH) is one of the most prevalent allergic skin diseases worldwide, with incidences varying from 3% to 60% in different countries^5^. IBH is characterized by severe pruritic dermatitis resulting from type I and type IV hypersensitivity reactions to salivary allergens, primarily from biting midges belonging to the genus *Culicoides*^5–7^. Previous studies of lesional skin in IBH have identified keratinocyte barrier dysfunction, increased cytokine expression, and accumulation of various immune cells, mainly mast cells, eosinophils, and Th2-cells^4,6,8–10^. These insights into IBH pathogenesis have largely been derived from histological analyses, immunological assays or bulk transcriptomic approaches. Treatment options for IBH are limited, and the disease significantly compromises animal welfare, diminishes performance capacity, and imposes substantial economic burdens through veterinary intervention costs^5,11^.

A deeper understanding of the pathophysiological mechanisms underlying IBH is essential for developing more effective therapeutic strategies. However, identifying disease-associated cellular responses requires a detailed characterization of normal equine skin as a reference state. To our knowledge, the cellular composition of equine skin has not yet been characterized at single-cell resolution, although similar studies have been performed for human skin^12,13^.

The overall aim of this study was to generate and validate a single-cell transcriptomic dataset of normal equine skin as a reference resource for future studies of equine skin biology and disease. Specifically, our objectives were to (i) generate high-quality single-cell RNA sequencing data from the skin of clinically healthy horses; (ii) assess dataset quality and cellular representation using established quality-control and annotation procedures; and (iii) provide raw and processed data, metadata, and analysis workflows to enable reuse by the research community.

To achieve these objectives, skin biopsies were obtained from the mane region of two clinically healthy horses during a period of minimal Culicoides activity and analysed using the Singleron Biotechnology platform. The resulting dataset comprises 85,574 high-quality transcriptomes resolving 22 distinct cell populations, together with fully documented processing workflows, quality control metrics and annotation criteria. This resource provides a single-cell reference dataset for normal equine skin to support future investigations into dermatological diseases, immune dysfunction, and cross-species biology.

## Methods

### Ethical statement

The study was conducted in compliance with the EU Directive 2010/63/EU for animal experiments and approved by the local ethical committee (Uppsala djurförsöksetiska nämnd; Dnr 5.8.18-06953/2024).

### Sample Collection

Skin biopsies were collected from two clinically healthy, privately owned mares housed in the same stable in Uppland, Sweden, out of season in October 2024: a 24-year-old Dutch Warmblood (donor C_1) and a 25-year-old Welsh pony (donor C_2). A single 6-mm skin biopsy was collected from the base of the mane of each mare using a biopsy punch following sedation with detomidine/butorphanol and local anaesthesia with mepivacaine. Biopsies were immediately placed in phosphate-buffered saline and maintained on ice until tissue processing.

### Tissue Dissociation and Single Cell Isolation

**Fig. 1:**
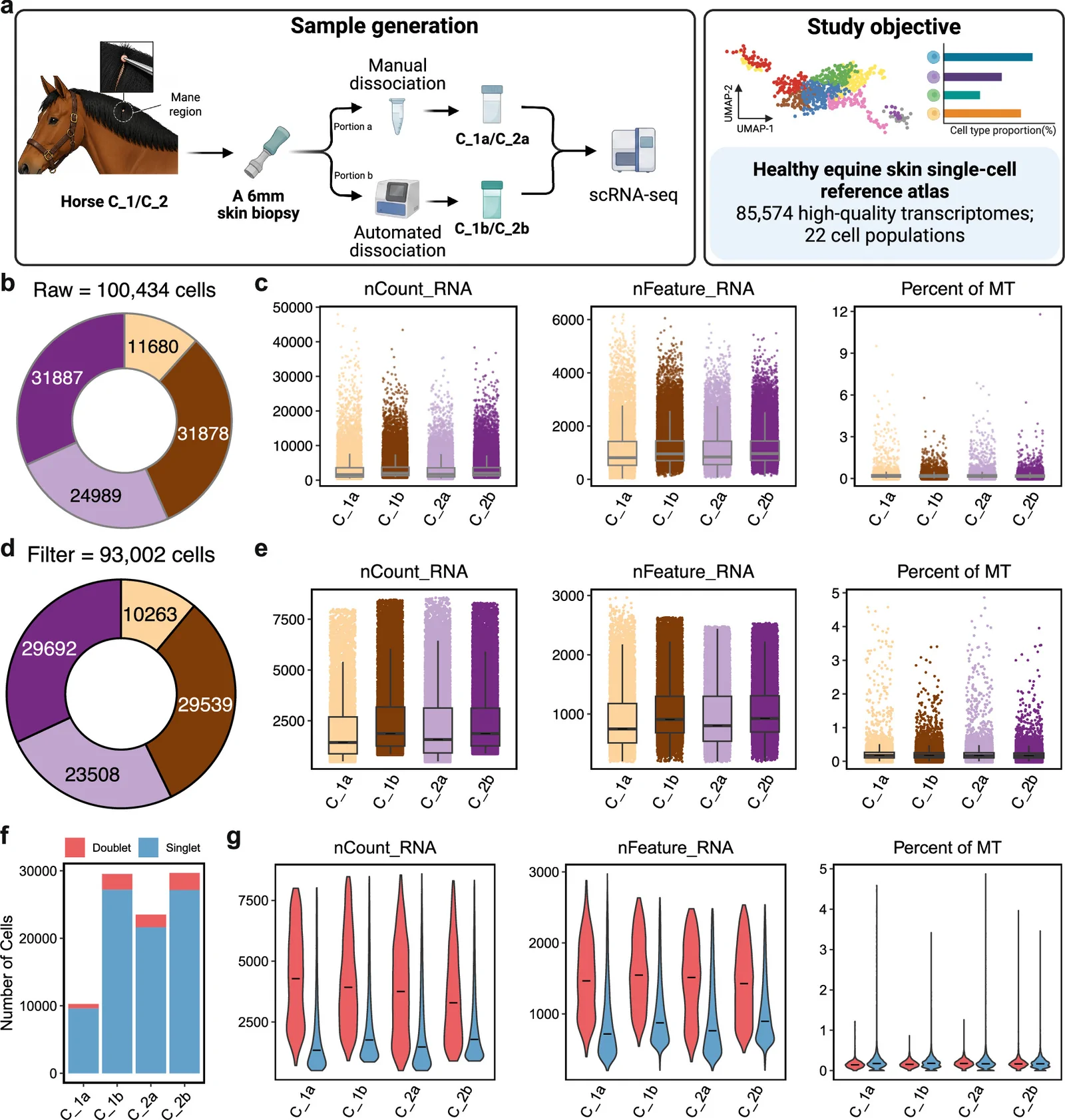
Quality control assessment and doublet detection in single-cell RNA sequencing dataset. (**a**) Experimental design and sample processing workflow for single-cell RNA sequencing of healthy equine skin. One skin biopsy was collected from each of two healthy horses (C_1 and C_2). Each biopsy was divided into two portions (**a** and **b**) and processed independently using either manual (**a**) or automated (**b**) tissue dissociation, generating four samples: C_1a, C_1b, C_2a, and C_2b. Each sample was processed independently for scRNA-seq. (**b,c**) Pre-filtering quality control overview. (**b**) Donut chart illustrating the total number and per-sample distribution of 100,434 captured barcodes across samples C_1a, C_1b, C_2a, and C_2b. (**c**) Box plots depicting the per-sample distributions of total RNA counts per cell (nCount_RNA), number of detected genes per cell (nFeature_RNA), and mitochondrial gene expression percentage (Percent of MT) prior to quality control filtering. (**d,e**) Post-filtering quality control overview. (**d**) Donut chart showing the total number and per-sample composition of 93,002 high-quality cells retained following application of adaptive, sample-specific filtering thresholds. (**e**) Box plots displaying the corresponding per-sample distributions of nCount_RNA, nFeature_RNA, and Percent of MT after removal of low-quality barcodes. (**f**) Bar chart summarizing the absolute number of computationally predicted doublets and singlets per sample following DoubletFinder-based detection. (**g**) Violin plots comparing the per-sample distributions of nCount_RNA, nFeature_RNA, and Percent of MT between predicted doublets and singlets.

One skin biopsy was collected from each of two healthy horses (C_1 and C_2). Each biopsy was divided into two portions (a and b), which were processed independently using two different tissue dissociation procedures. Portion a underwent manual dissociation, whereas portion b underwent automated dissociation. The resulting four samples: C_1a (Horse C_1, manual dissociation), C_1b (Horse C_1, automated dissociation), C_2a (Horse C_2, manual dissociation), and C_2b (Horse C_2, automated dissociation) (Table 1, Fig. 1a). Each sample was subsequently processed independently for single-cell RNA sequencing. The biopsies were shipped at 4 °C in sCelLive^®^ tissue Preservation Solution (mat#1200050001) to Singleron Biotechnologies GmbH (Cologne, Germany) for tissue dissociation and single-cell RNA sequencing service. The tissues were processed into single cell suspensions using the sCelLive® Tissue Dissociation Kit (Skin) (cat# 1200500030, Singleron Biotechnologies GmbH, Cologne) according to the manufacturer’s instructions. Tissues were briefly washed once with HBSS (Gibco, cat. no. 24020117), resuspended in 1.8 mL sCelLiVE® Tissue Dissociation Mix (Skin), and minced into 1–2 mm^2^ pieces using ophthalmic scissors. Later, for each horse, one portion (a) was subjected to manual dissociation, while the other portion (b) was subjected to automated dissociation. Automated dissociation was performed using Singleron PythoN Junior® Automated Tissue Dissociation System (MD1102001, Singleron Biotechnologies GmbH, Cologne) with the following program setting: 37 °C, 30 rpm, 15 s counterclockwise rotation, 10 s clockwise rotation, 180 cycles. Manual dissociation was performed by incubating the samples at 37 °C for 90 minutes with continuous agitation at 350 rpm, with vigorous pipetting every 10 minutes. Dissociation progress was monitored by microscopy every 30 minutes. Resulting suspensions were filtered sequentially through 100 µm and 40 µm strainers, then washed and resuspended in PBS supplemented with 0.04% bovine serum albumin. Cell numbers and viability were measured using Acridine Orange/Propidium Iodide staining with the Luna FX7 automated cell counter (Logos Biosystems, Villeneuve d’Ascq, France).

**Table 1:** Overview of sample identity, collection, and processing parameters.

Sample ID	Age	Sex	Breed	Donor ID	Dissociation method	Collection date	Tissue weight (mg)	Viability (%)
C_1a	24	mare	Dutch Warmblood	C_1	Manual	2024-10-30	130	87
C_1b	24	mare	Dutch Warmblood	C_1	Automated	2024-10-30	117	82
C_2a	25	mare	Welsh pony	C_2	Manual	2024-10-30	107	74
C_2b	25	mare	Welsh pony	C_2	Automated	2024-10-30	109	82

### Single-Cell RNA-seq Library Preparation

To partition the single cells, 100 µl of each cell suspension, containing on average 22,500 cells (manual dissociation) and 60,000 cells (automated dissociation), were loaded onto the SCOPE^®^-chip from the GEXSCOPE^®^ Single Cell RNA Library Kit V2 (cat# 4180011, Singleron Biotechnologies). Paramagnetic beads conjugated with oligonucleotide barcodes and unique molecular identifiers (UMIs) were loaded onto the SCOPE^®^-chip to capture mRNA and label transcripts during reverse transcription. cDNA amplification and library construction were carried out according to the manufacturer’s instructions. The libraries were sequenced on an Illumina NovaSeq X with a paired-end 150-bp approach by Macrogen (Amsterdam). Sequencing reads were demultiplexed using Illumina BaseCloud.

### Transcriptome Preprocessing

Raw sequencing reads were processed into gene expression matrices using CeleSCOPE^®^ version 2.0.7 (www.github.com/singleron-RD/CeleScope; Singleron Biotechnologies). FASTQ file barcode chemistry determination and barcode correction were performed before demultiplexing, and gene counts were generated using the STARSolo single-cell pipeline with STAR version 2.7.11a [https://github.com/alexdobin/STAR] and STARsolo [https://github.com/cellgeni/STARsolo]. The Genome EquCab3.0 NCBI RefSeq assembly (GCF_002863925.1) was used for mapping and annotation. Cell calling was based on bimodal modeling^14,15^.

### Single-Cell RNA Sequencing Data Quality Control and Preprocessing

Processed single-cell RNA sequencing (scRNA-seq) matrix was analysed using the Seurat (v5. x) package in R^16^. To ensure robust removal of low-quality barcodes while preserving biologically meaningful cellular heterogeneity, quality control (QC) thresholds were defined adaptively on a per-sample basis across the four samples analyzed. Three primary QC metrics were evaluated: total RNA counts per cell (*nCount_RNA*), number of detected genes per cell (*nFeature_RNA*), and the proportion of reads mapping to mitochondrial genes (*percent.mt*).

For *nCount_RNA*, thresholds were determined using a median absolute deviation (MAD)-based approach, with the lower bound defined as the maximum of *(median − 3 × MAD)* and 500 counts, and the upper bound defined as *(median + 5 × MAD)*. For *nFeature_RNA*, a fixed lower bound of 200 genes was applied to remove empty droplets and transcriptionally inactive barcodes. The upper bound was defined as the minimum of the sample-specific 95th percentile and an absolute ceiling of 6,000 genes to exclude potential multiplets. Mitochondrial content thresholds were also determined adaptively: samples with a median mitochondrial percentage below 1% were filtered using a 5% cutoff, whereas samples with higher baseline mitochondrial expression used a threshold defined as the minimum of *(median + 3 × MAD)* and 20%. Following QC filtering, computational doublet detection was performed using DoubletFinder^17^. For each sample, the expected doublet rate was estimated proportionally based on the number of recovered cells using a linear model, reflecting the increased probability of co-encapsulation at higher cell densities, with a maximum cap of 10%. The optimal pK parameter was determined empirically via parameter sweep and BCmvn maximization, and homotypic doublet proportions were estimated from preliminary cluster annotations to adjust the expected doublet number. Predicted doublets were removed prior to downstream analyses.

### Dimensionality Reduction, Clustering, and Cell Type Annotation

Following QC and doublet removal, 85,574 singlet cells were retained. Ribosomal (RPS, RPL) and mitochondrial (MT-) transcripts were excluded prior to normalization. Data were log-normalized using Seurat’s *NormalizeData*, and the top highly variable genes were identified with *FindVariableFeatures*. Scaled expression values were computed via *ScaleData*, regressing out percent mitochondrial content and total RNA counts. Principal component analysis (PCA) on variable genes captured major sources of variation, and batch effects were corrected with Harmony^18^. Integrated embeddings were visualized with uniform manifold approximation and projection (UMAP), and clusters were defined on a shared nearest-neighbour graph using the Louvain algorithm. Cell identities were assigned based on cluster-specific marker genes (Data S1) identified via Wilcoxon rank-sum testing and validated against canonical literature markers from mammalian skin single-cell atlases^19,20^.

### Pathway Enrichment Analysis

Cell–type–specific pathway enrichment analysis was performed using the cluster Profiler package in R^21^. For each annotated cell type, differentially expressed genes identified using the Wilcoxon rank-sum test (log2 fold change > 0.2 & adjusted p < 0.05, Bonferroni correction) were subjected to Kyoto Encyclopedia of Genes and Genomes (KEGG) pathway over-representation analysis^18^. Adjusted p-values were calculated using the Benjamini–Hochberg false discovery rate correction method.

### Functional Gene Module Scoring Analysis

To characterize the activity of biologically coherent functional programs at single-cell resolution, curated gene module scores were calculated using AUCell^4^. Gene modules were defined based on literature-supported gene sets representing 12 distinct functional activities^22–24^.

## Data Records

The raw FASTQ files from the single-cell RNA sequencing of healthy equine skin have been deposited in the NCBI Sequence Read Archive under BioProject accession number PRJNA1441223 (https://www.ncbi.nlm.nih.gov/bioproject/PRJNA1441223) and SRA Study accession number SRP690566 (https://identifiers.org/insdc.sra:SRP690566)^25^. The dataset comprises four SRA experiments: SRX32874927 (C_1a; SRR38022133), SRX32874928 (C_1b; SRR38022132), SRX32874929 (C_2a; SRR38022131), and SRX32874930 (C_2b; SRR38022130). Skin biopsies were collected from the mane region of two clinically healthy horses. One biopsy from each horse was divided into two portions, which were independently processed using either manual or automated tissue dissociation, resulting in four scRNA-seq samples. The processed and integrated RDS file, together with supplementary materials, including three Supplementary Data files and two Supplementary Figures, has been deposited in Figshare and is available at https://doi.org/10.6084/m9.figshare.31980441^26^.

## Technical Validation

### Quality Control Filtering Yields a High-Confidence Single-Cell Dataset

A total of 100,434 cells were initially captured across four control samples (Fig. 1b,c). Adaptive per-sample QC filtering based on nCount_RNA, nFeature_RNA, and percent mitochondrial content retained 93,002 high-quality cells, corresponding to an overall retention rate of 92.6% (Fig. 1d, Table 2). Retention was consistent across samples: C_1a, 87.89% (10,263/11,680); C_1b, 92.66% (29,539/31,878); C_2a, 94.08% (23,508/24,989); and C_2b, 93.12% (29,692/31,887). Post-QC distributions of nCount_RNA (5th–95th percentile: C_1a 569–6,149; C_1b 932–6,347; C_2a 570–6,009; C_2b 959–6,050), nFeature_RNA (C_1a 356–2,067; C_1b 538–2,124; C_2a 375–2,095; C_2b 554–2,045), and Percent of MT (0–~0.4%) were highly consistent, reflecting uniform data quality and minimal batch effects (Fig. 1d,e**;** Table 2).

**Table 2:** Single-Cell RNA-seq Quality Control Summary Before and After Filtering.

Sample	Pre-QC				Post-QC				Retained (%)
	Number of cells	n_Count (5–95%)	n_Feature (5–95%)	Percent of MT (5–95%)	Number of cells	n_Count (5–95%)	n_Feature (5–95%)	Percent of MT (5–95%)	
C_1a	11,680	(573, 11957)	(344, 3144)	(0, 0.4612)	10,263	(569, 6149)	(356, 2067)	(0, 0.4790)	87.89
C_1b	31,878	(937, 9437)	(542, 2637)	(0, 0.4076)	29,539	(932, 6347)	(538, 2124)	(0, 0.4129)	92.66
C_2a	24,989	(572, 7979)	(375, 2483)	(0, 0.4252)	23,508	(570, 6009)	(375, 2095)	(0, 0.4329)	94.08
C_2b	31,887	(964, 9056)	(557, 2538)	(0, 0.3912)	29,692	(959, 6050)	(554, 2045)	(0, 0.3981)	93.12
Overall	100,434				93,002				92.6

### Doublet Removal Further Refines Cell Quality

Computational doublet calling identified 7,428 putative doublets (7.99% overall; Fig. 1f, Table 3), with per-sample counts: C_1a 661 (6.44%), C_1b 2,345 (7.94%), C_2a 1,882 (8.01%), C_2b 2,540 (8.55%). Doublets exhibited elevated nCount_RNA (e.g., C_1b 1,744–7,471 vs. singlets 564–5716.85) and nFeature_RNA (754–2,343 vs. 353–2,000.95), consistent with transcriptomic inflation from co-encapsulated cells, while mitochondrial fractions remained low (0–~0.4%), indicating unbiased doublet classification. Excluding doublets yielded 85,574 high-confidence singlets for downstream analysis (Fig. 1f–g**;** Table 3).

**Table 3:** Doublet and Singlet Detection Summary in Single-Cell RNA-seq.

Sample	Doublet				Singlet				Retained (%)
	Number of cells	n_Count (5–95%)	n_Feature (5–95%)	Percent of MT (5–95%)	Number of cells	n_Count (5–95%)	n_Feature (5–95%)	Percent of MT (5–95%)	
C_1a	661	(1744, 7471)	(745,2343)	(0.0355, 0.2875)	9602	(564, 57167)	(353, 2001)	(0, 0.4874)	93.56
C_1b	2345	(1619, 7673)	(812, 2413)	(0.0443, 0.3159)	27194	(926, 6067)	(534, 2029)	(0, 0.4196)	91.38
C_2a	1882	(1065, 7172)	(606, 2326)	(0.0463, 0.3436)	21626	(564, 5747)	(372, 2031)	(0, 0.4411)	91.99
C_2b	2540	(1192, 6998)	(673, 2306.05)	(0.0351, 0.3582)	27152	(954, 5864)	(549, 1978)	(0, 0.4010)	91.43
Overall	7428				85,574				92.09

### Single-Cell RNA Sequencing Resolves the Cellular Landscape of Equine Skin

**Fig. 2:**
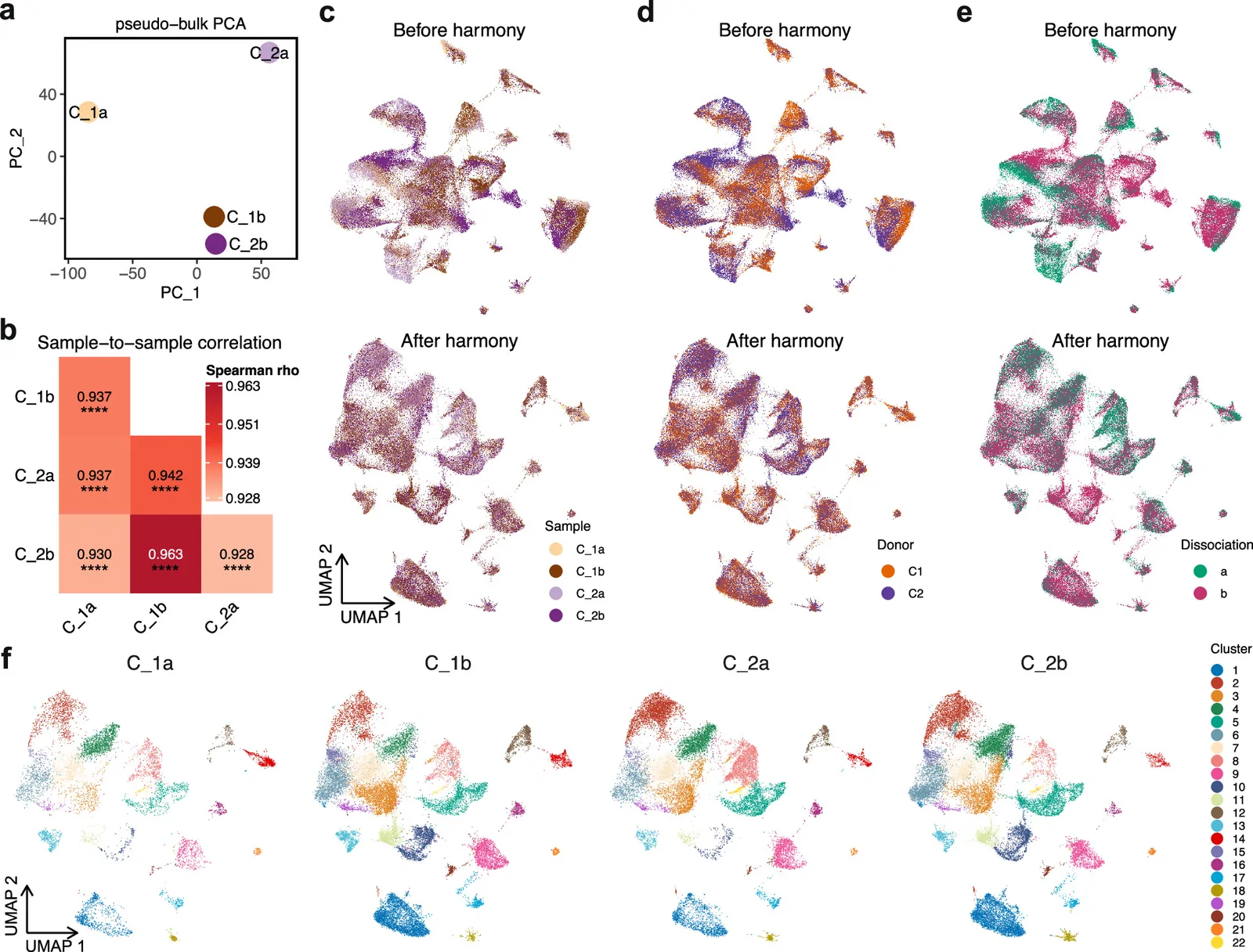
Sample-level relationships, batch correction, and marker-based validation of annotated cell populations. (**a**) Pseudo-bulk PCA of the four samples, based on aggregated gene expression profiles. C_1a and C_1b represent portions of the same biopsy from horse C_1 processed using manual (**a**) and automated (**b**) dissociation, respectively. C_2a and C_2b were generated similarly from a single biopsy from horse C_2. (**b**) Sample-to-sample correlation matrix of pseudo-bulk expression profiles (****p < 0.0001). (**c**–**e**) UMAP projections before (top) and after (bottom) Harmony batch correction, colored by (**c**) sample, (**d**) donor, and (**e**) dissociation method, illustrating removal of sample-level technical variation and confirming that donor- and dissociation-associated structure was also resolved following integration. (**f**) UMAP projections split by sample (C_1a: 9602; C_1b: 27194; C_2a: 21626; C_2b: 27152 cells) after Harmony integration, colored by cluster identity (clusters 1–22), showing that all 22 clusters were represented across all four samples following batch correction.

Pseudo-bulk PCA showed the two dissociation replicates (“a” vs. “b”) separating along PC_2, with C_1b and C_2b clustering closely together at negative PC_2 values, whereas C_1a and C_2a, despite both falling at positive PC_2 values, were widely separated from one another along PC_1 and did not cluster together (Fig. 2a). Spearman correlation analysis of pseudo-bulk profiles showed uniformly high pairwise correlations across all sample combinations (ρ = 0.928–0.963, all p < 0.0001) (Fig. 2b), with the highest value observed between C_1b and C_2b (ρ = 0.963), consistent with their proximity in PCA space; however, all other pairwise correlations fell within a similarly narrow range, indicating that overall transcriptional profiles were highly similar across all four samples regardless of donor or dissociation methods. Given this sample-level variation, Harmony integration was performed using sample identity as the correction variable prior to downstream clustering. Before integration, cells showed visible clustering by sample (Fig. 2c), with corresponding structure also apparent when colored by donor (Fig. 2d) and dissociation method (Fig. 2e). Following integration, cells from all four samples intermixed extensively within shared clusters (Fig. 2c), and this mixing was similarly evident when cells were colored by donor (Fig. 2d) or dissociation method (Fig. 2e), confirming that Harmony correction on sample identity was sufficient to resolve technical variation associated with both underlying factors. Consistent with this, when the integrated UMAP was split by individual sample, all 22 clusters were represented across all four samples (C_1a, C_1b, C_2a, C_2b) (Fig. 2f), rather than being restricted to particular samples, donors, or dissociation methods.

**Fig. 3:**
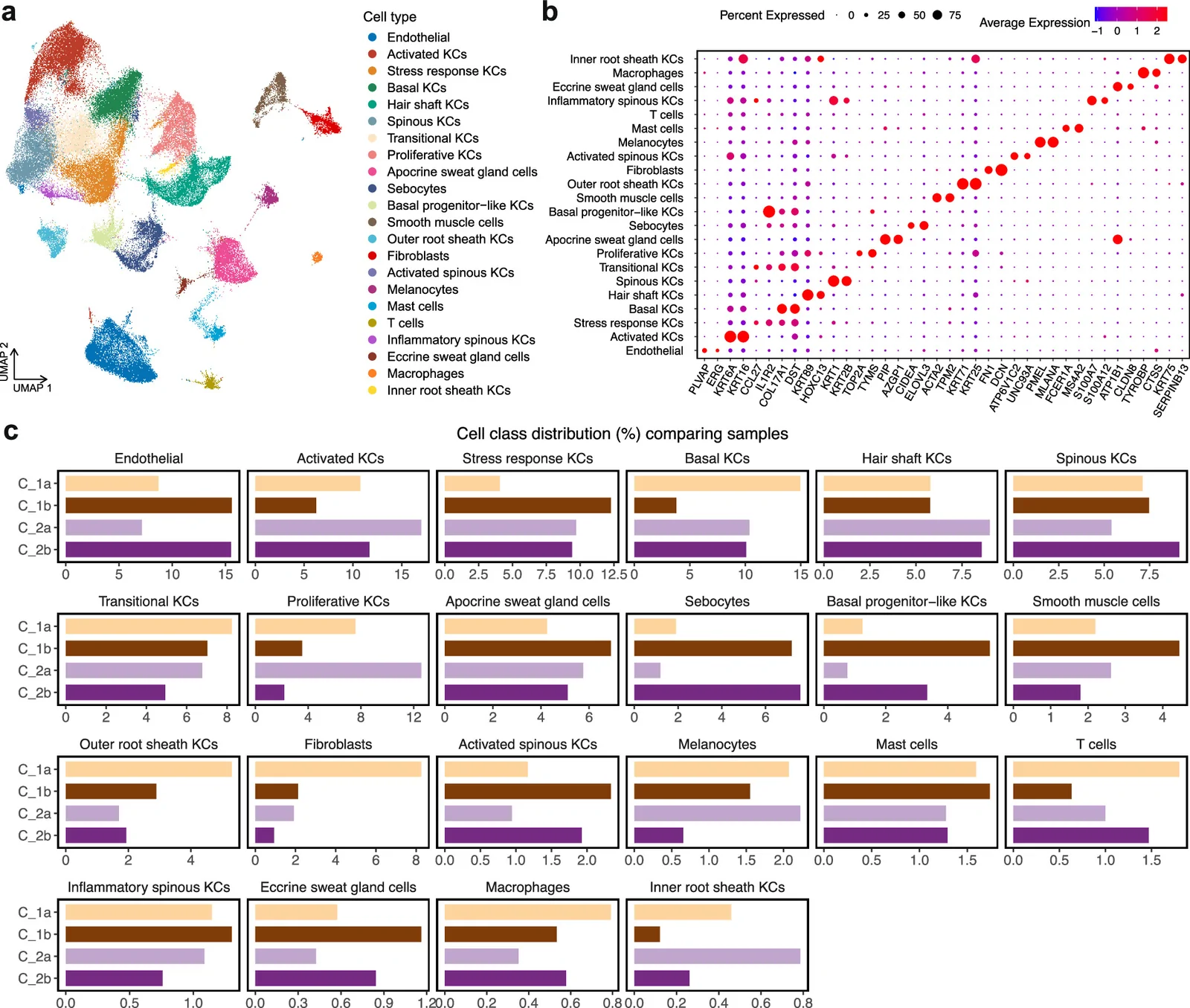
Cell type identity, spatial organization, and proportional abundance across replicate skin samples. (**a**) UMAP projection of 85,574 cells pooled from four samples, with cells colored according to their annotated cell type (C_1a: 9602; C_1b: 27194; C_2a: 21626; C_2b: 27152 cells). (**b**) Dot plot illustrating the expression patterns of curated cell-type-specific marker genes across the 22 identified cell populations, with dot size representing the percentage of cells expressing each gene and color representing average scaled expression level. (**c**) Bar plots showing the percentage abundance of each individual cell type in each of the four samples, enabling direct quantitative comparison of cell type frequency between samples. C_1a and C_1b represent portions of the same biopsy from horse C_1 processed using manual (**a**) and automated (**b**) dissociation, respectively. C_2a and C_2b were generated similarly from a single biopsy from horse C_2.

Unsupervised graph-based clustering of 85,574 singlets with UPlease move the last sentence of the Figure 2 legend to the Results section, in the following paragraph.MAP dimensionality reduction resolved 22 transcriptionally distinct cell populations spanning the major cellular compartments of the skin (Fig. 3a), with cluster identities assigned by integrating differentially expressed genes with canonical cell type markers and validated via curated feature plots (Fig. 3b, Figure S1).The annotated populations encompassed the major cell types and cellular states expected in equine skin. Keratinocyte subpopulations spanning canonical differentiation states (basal, basal progenitor-like, transitional, and spinous), together with proliferative, activated KCs, stress-response, and activated/inflammatory spinous states, constituted the dominant cellular fraction (55.28% overall; range 49.33–64.61%). The proliferative KC cluster was defined by canonical cell-cycle markers (TOP2A, PCNA, TYMS, RRM2, UBE2C), consistent with the actively cycling basal/transit-amplifying compartment required for physiological epidermal renewal, and similar populations have been reported in normal human epidermis^27,28^. Activated KCs were defined by KRT6A, KRT6B, KRT6C, KRT16, and KRT17. While these keratins have historically been described as “hyperproliferative” due to their induction in psoriasis and wound healing, recent single-cell studies indicate they more broadly reflect epithelial activation, differentiation, and inflammatory signaling rather than active proliferation^29^. Low-level expression of these keratins has also been reported in clinically healthy human and equine skin^6,29^, consistent with their presence as part of normal keratinocyte heterogeneity^12^. Stress-response KCs were defined by immediate-early response genes (EGR1, DUSP1, ATF3, JUN, JUND, NR4A1, IER2, HSPA6, DNAJB1, GADD45A/B, NFKBIA). This population did not show a consistent association with dissociation method within donors (C_1a: 4.07% vs. C_1b: 12.26%; C_2a: 9.70% vs. C_2b: 9.40%), suggesting the observed variation is unlikely to be fully explained by dissociation method alone^30,31^.

Adnexal populations, including apocrine and eccrine sweat gland cells, hair shaft KCs, sebocytes, and inner and outer root sheath KCs, accounted for 22.17% of cells overall (range 18.22–24.30%), confirming preservation of skin appendage-associated cell types. Non-epithelial populations encompassed three functional compartments: vascular and pigment cells, including endothelial cells and melanocytes (14.15%; range 9.27–17.14%); stromal cells, including fibroblasts and smooth muscle cells (5.27%; range 2.73–10.46%); and immune cells, including T cells, mast cells, and macrophages (3.11%; range 2.63–4.19%) — the latter consistent with the low-grade immune surveillance characteristic of homeostatic skin.

Comparison of cell type proportions across the four samples showed that nine of the 22 annotated populations — transitional KCs, activated spinous KCs, melanocytes, eccrine sweat gland cells, inner root sheath KCs, mast cells, macrophages, inflammatory spinous KCs, and T cells — showed minimal variation (<2 percentage points) both between dissociation methods within each donor and between donors, and were considered broadly comparable across all four samples (Fig. 3c, Data S2).

Four cell types were consistently associated with dissociation method rather than donor identity (Figure S2b): endothelial cells (C_1a: 8.71%, C_1b: 15.59%; C_2a: 7.14%, C_2b: 15.52%), sebocytes (C_1a: 1.91%, C_1b: 7.20%; C_2a: 1.19%, C_2b: 7.59%), and basal progenitor-like KCs (C_1a: 1.25%, C_1b: 5.36%; C_2a: 0.76%, C_2b: 3.33%) were consistently higher with automated dissociation (“b”), while proliferative KCs (C_1a: 7.59%, C_1b: 3.55%; C_2a: 12.57%, C_2b: 2.19%) were consistently higher with manual dissociation (“a”); all four showed comparable proportions between donors once aggregated across method. Activated KCs showed a mixed pattern, consistently higher under manual dissociation within each donor (C_1a: 10.76%, C_1b: 6.24%; C_2a: 17.01%, C_2b: 11.69%) but also higher overall in C_2 than in C_1 (14.35% vs. 8.50%, averaged across method), suggesting independent contributions from both dissociation method and donor identity. One cell type, hair shaft KCs, was associated with donor identity rather than dissociation method (Figure S2a): its proportion was nearly identical between methods within each donor (C_1a: 5.78%, C_1b: 5.77%; C_2a: 9.01%, C_2b: 8.57%) but differed by 3 percentage points between donors when averaged across method (5.77% in C_1 vs. 8.79% in C_2).

The remaining seven cell types — basal KCs, fibroblasts, outer root sheath KCs, stress response KCs, apocrine sweat gland cells, smooth muscle cells, and spinous KCs — showed proportional differences that were not consistently attributable to either dissociation method or donor identity, but instead appeared to be driven by a single sample. Basal KCs, fibroblasts, and outer root sheath KCs were each disproportionately elevated in C_1a relative to the other three samples (14.96%, 8.26%, and 5.31%, respectively), while stress response KCs, apocrine sweat gland cells, and smooth muscle cells were each elevated specifically in C_1b (12.26%, 6.90%, and 4.45%, respectively), and spinous KCs were elevated specifically in C_2b (9.14%). These patterns are more likely to reflect local tissue heterogeneity within individual biopsy portions than a systematic effect of the dissociation method or donor.

Taken together, a large subgroup of most annotated populations (9 of 22) showed consistent proportions across all four samples, four were associated primarily with the dissociation method, one with donor identity, one with both, and the remaining seven showed sample-specific variation not attributable to either factor. Given the small sample size (n = 2/donor), these patterns should be interpreted descriptively rather than as statistically confirmed effects.

### Cell Type-Specific Pathway Enrichment Reveals Distinct Functional Identities

**Fig. 4:**
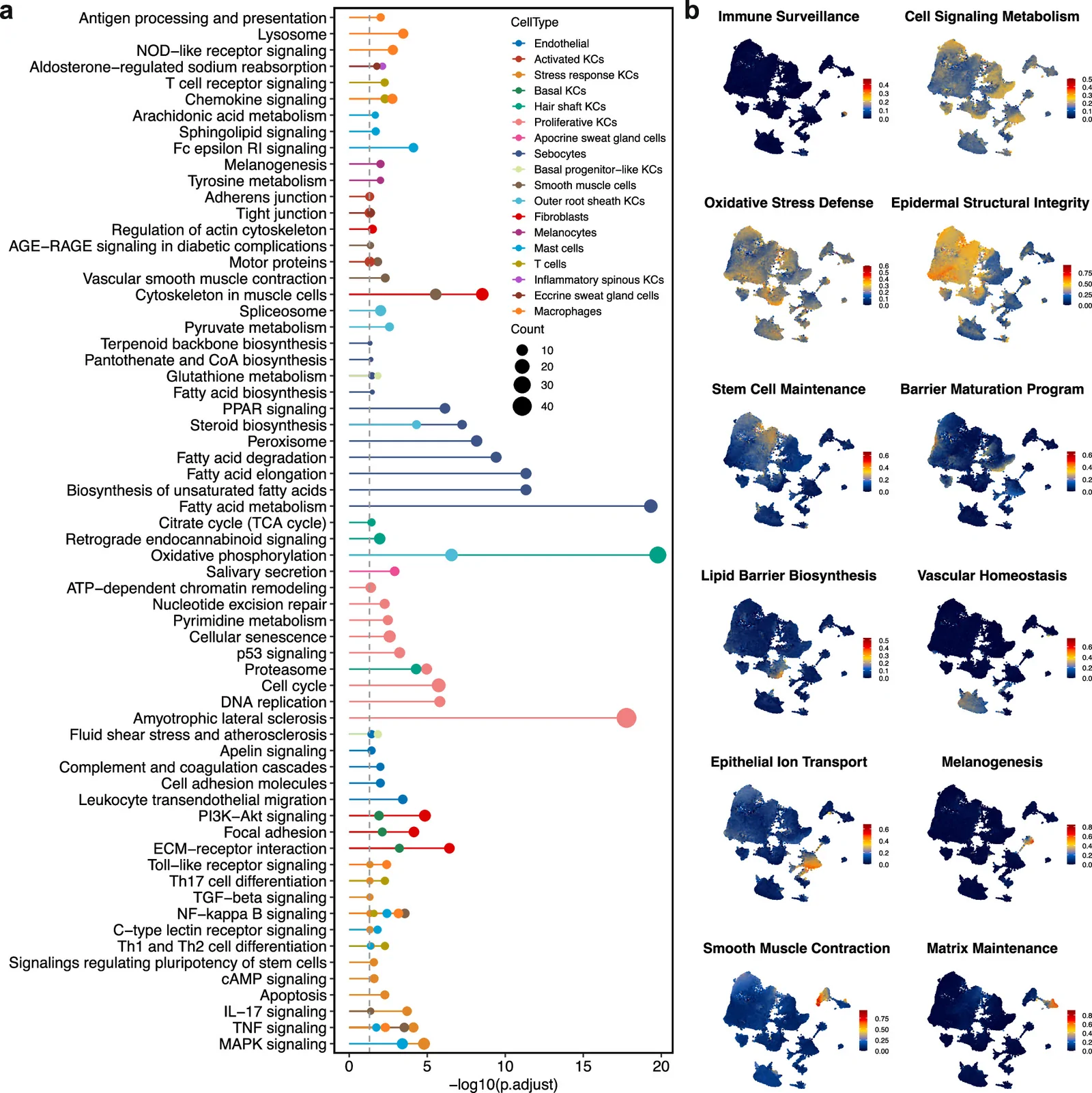
Cell type-specific pathway enrichment and functional module analysis. (a) Dot plot showing significantly enriched KEGG pathways across different cell types derived from cell type-specific differentially expressed genes identified across all 85,574 cells pooled from 4 samples. The x-axis represents the statistical significance of enrichment (-log10(adjusted p-value)), dot size indicates the number of genes (count) associated with each pathway, and dot color represents different cell types as indicated in the legend. (b) UMAP plots show the distribution and expression levels of 12 functional gene modules, projected onto the same batch-corrected dataset of 85,574 cells (all four samples, both 2 donors and both dissociation methods) shown in Fig. 3a. Each module represents a specific biological process. Color intensity, ranging from blue to red, indicates the module score, where red represents higher (activated) module activity and blue represents lower module activity. The spatial distribution of module scores reveals cell type-specific functional specialization and biological programs active in different cell populations.

To validate the accuracy of cell type annotation, KEGG pathway enrichment analysis was performed on cell type-specific differentially expressed genes (Fig. 4a, Data S3), confirming highly cell type-specific signatures consistent with the known specialized roles of each population.

Within the keratinocyte compartment, basal KCs were enriched in ECM–receptor interaction and focal adhesion pathways, supporting their role in basement membrane anchoring, while activated KCs showed enrichment in tight junction and adherens junction signaling, indicative of barrier integrity maintenance. Proliferative KCs were most prominently associated with DNA replication, cell cycle, and p53 signaling (−log_10_ adjusted *p* > 5), characteristic of active mitotic activity. Stress response KCs were enriched in MAPK, TNF, and IL-17 signaling and apoptosis, matching their inflammatory activation and stress-induced cell death. Hair shaft and outer root sheath KCs both showed strong oxidative phosphorylation enrichment, pointing to high mitochondrial demands during terminal differentiation. Sebocytes exhibited the most metabolically specialized profile, with fatty acid metabolism and biosynthesis of unsaturated fatty acids among the most significantly enriched pathways (−log_10_ adjusted *p* > 10), in line with their dedicated role in sebum synthesis.

Among immune populations, macrophages were enriched in NF-κB, NOD-like receptor, Toll-like receptor, and chemokine signaling, alongside antigen processing and presentation, reflecting innate immune sensing and inflammatory coordination. Mast cells were most prominently enriched in Fc epsilon RI signaling – the canonical IgE-mediated allergic activation pathway –alongside MAPK signaling and arachidonic acid metabolism. T cells showed enrichment in T cell receptor signaling, Th1/Th2 and Th17 differentiation, and chemokine signaling, consistent with adaptive effector functions. Endothelial cells were enriched in leukocyte transendothelial migration and in the complement and coagulation cascades, in line with their roles in immune trafficking and vascular inflammation.

Stromal populations exhibited complementary profiles: smooth muscle cells were enriched in cytoskeletal and vascular smooth muscle contraction pathways alongside NF-κB and TNF signaling, while fibroblasts showed enrichment in ECM–receptor interaction, PI3K–Akt signaling, and focal adhesion, aligning with matrix remodeling and mechano-survival signaling in the dermal microenvironment.

### Functional Module Analysis Identifies Cell Type-Specific Biological Programs

To further validate functional coherence across the annotated cell population, module scores for 12 curated biological programs were projected onto the UMAP embedding (Fig. 4b). Distinct spatial patterns of module activity were observed, with each functional program enriched in transcriptionally and anatomically coherent cell populations, further supporting the accuracy of cell type annotations.

The Immune Surveillance module showed uniformly low activity across all clusters, consistent with the absence of overt immune activation in normal skin tissue. In contrast, the Oxidative Stress Defense module displayed widespread activation across nearly all cell types, as expected for the constitutive antioxidant defenses required to maintain redox homeostasis in environmentally exposed skin. The Cell Signaling and Metabolism module was similarly widespread but showed peak scores in hair shaft KCs, activated KCs, proliferative KCs, and apocrine sweat gland cells, consistent with elevated metabolic demands during epithelial proliferation, terminal differentiation, and glandular secretion.

Epidermal functional modules showed clear lineage-specificity for keratinocytes. Epidermal Structural Integrity and Barrier Maturation modules were concentrated within keratinocyte clusters, while the Lipid Barrier Biosynthesis module was strongly and selectively enriched in sebocytes. The Stem Cell Maintenance module was highest in basal KCs, as expected for their progenitor identity.

Cell type-restricted enrichment was equally evident across non-epithelial populations. The Epithelial Ion Transport module was confined to apocrine sweat gland cells; the Vascular Homeostasis and Smooth Muscle Contraction modules were exclusively active in endothelial cells and smooth muscle cells, respectively; the Melanogenesis module was restricted to melanocytes; and the Matrix Maintenance module was predominantly active in fibroblasts.

Collectively, these module activity profiles recapitulate the known biology of each annotated population, providing orthogonal functional validation of the cell type assignments and confirming the biological coherence of the dataset. Together with the high cell recovery rate, low doublet contamination, robust cell-type-specific marker expression, and functionally coherent pathway enrichment patterns, these demonstrate the technical quality and biological validity of this single-cell dataset, confirming its suitability for downstream comparative and integrative analyses.

## Data Availability

The raw FASTQ files from the single-cell RNA sequencing of healthy equine skin have been deposited in the NCBI Sequence Read Archive under BioProject accession number PRJNA1441223 (https://www.ncbi.nlm.nih.gov/bioproject/PRJNA1441223) and SRA Study accession number SRP690566 (https://identifiers.org/insdc.sra:SRP690566)^25^. The dataset comprises four SRA experiments: SRX32874927 (C_1a; SRR38022133), SRX32874928 (C_1b; SRR38022132), SRX32874929 (C_2a; SRR38022131), and SRX32874930 (C_2b; SRR38022130). Skin biopsies were collected from the mane region of two clinically healthy horses. One biopsy from each horse was divided into two portions, which were independently processed using either manual or automated tissue dissociation, resulting in four scRNA-seq samples. The processed and integrated RDS file, together with supplementary materials, including three Supplementary Data files and two Supplementary Figures, has been deposited in Figshare and is available at https://doi.org/10.6084/m9.figshare.31980441^26^.

## Code availability

The code used for analysis and the figure in this study is available in the GitHub repository: https://github.com/Molmed/Equine_skin_scSeq.

## References

[1] Scott, D. W. & Miller, W. H. in *Equine Dermatology (Second Edition)* (eds Danny W. Scott & William H. Miller) 1–34 10.1016/B978-1-4377-0920-9.00001-9 (W.B. Saunders, 2011).

[2] Sloet van Oldruitenborgh-Oosterbaan, M. M. & Grinwis, G. C. M. Basics of equine dermatology. *Equine Veterinary Education* **28**, 520–529 10.1111/eve.12444 (2016).

[3] Jørgensen, E., Lazzarini, G., Pirone, A., Jacobsen, S. & Miragliotta, V. Normal microscopic anatomy of equine body and limb skin: A morphological and immunohistochemical study. *Ann Anat* **218**, 205–212 10.1016/j.aanat.2018.03.010 (2018). 29730469

[4] van der Haegen, A. *et al*. Immunoglobulin-E-bearing cells in skin biopsies of horses with insect bite hypersensitivity. *Equine Vet J* **33**, 699–706 10.2746/042516401776249444 (2001). 11770993

[5] Marsella, R. *et al*. Equine allergic skin diseases: Clinical consensus guidelines of the World Association for Veterinary Dermatology. *Vet Dermatol* **34**, 175–208 10.1111/vde.13168 (2023). 37154488

[6] Cvitas, I. *et al*. Investigating the epithelial barrier and immune signatures in the pathogenesis of equine insect bite hypersensitivity. *PLoS One* **15**, e0232189 10.1371/journal.pone.0232189 (2020). PMC7188278 32343720

[7] Wagner, B. *et al*. IgE and IgG antibodies in skin allergy of the horse. *Vet Res* **37**, 813–825 10.1051/vetres:2006039 (2006). 16973120

[8] Heimann, M. *et al*. Skin-infiltrating T cells and cytokine expression in Icelandic horses affected with insect bite hypersensitivity: a possible role for regulatory T cells. *Vet Immunol Immunopathol* **140**, 63–74 10.1016/j.vetimm.2010.11.016 (2011). 21168921

[9] Fettelschoss-Gabriel, A. *et al*. Treating insect-bite hypersensitivity in horses with active vaccination against IL-5. *J Allergy Clin Immunol* **142**, 1194–1205.e1193 10.1016/j.jaci.2018.01.041 (2018). 29627082

[10] Jebbawi, F. *et al*. Cytokines and chemokines skin gene expression in correlation with immune cells in blood and severity in equine insect bite hypersensitivity. *Front Immunol* **15**, 1414891 10.3389/fimmu.2024.1414891 (2024). PMC11284025 39076967

[11] Jonsdottir, S. *et al*. New Strategies for Prevention and Treatment of Insect Bite Hypersensitivity in Horses. *Current Dermatology Reports* **8**, 303–312 10.1007/s13671-019-00279-w (2019).

[12] Cheng, J. B. *et al*. Transcriptional Programming of Normal and Inflamed Human Epidermis at Single-Cell Resolution. *Cell Rep* **25**, 871–883 10.1016/j.celrep.2018.09.006 (2018). PMC6367716 30355494

[13] Solé-Boldo, L. *et al*. Single-cell transcriptomes of the human skin reveal age-related loss of fibroblast priming. *Commun Biol* **3**, 188 10.1038/s42003-020-0922-4 (2020). PMC7181753 32327715

[14] Lun, A. T. L. *et al*. EmptyDrops: distinguishing cells from empty droplets in droplet-based single-cell RNA sequencing data. *Genome Biology* **20**, 63 10.1186/s13059-019-1662-y (2019). PMC6431044 30902100

[15] Griffiths, J. A., Richard, A. C., Bach, K., Lun, A. T. L. & Marioni, J. C. Detection and removal of barcode swapping in single-cell RNA-seq data. *Nature Communications* **9**, 2667 10.1038/s41467-018-05083-x (2018). PMC6039488 29991676

[16] Hao, Y. *et al*. Dictionary learning for integrative, multimodal and scalable single-cell analysis. *Nat Biotechnol* **42**, 293–304 10.1038/s41587-023-01767-y (2024). PMC10928517 37231261

[17] McGinnis, C. S., Murrow, L. M. & Gartner, Z. J. DoubletFinder: Doublet Detection in Single-Cell RNA Sequencing Data Using Artificial Nearest Neighbors. *Cell Syst* **8**, 329–337 e324 10.1016/j.cels.2019.03.003 (2019). PMC6853612 30954475

[18] Korsunsky, I. *et al*. Fast, sensitive and accurate integration of single-cell data with Harmony. *Nat Methods* **16**, 1289–1296 10.1038/s41592-019-0619-0 (2019). PMC6884693 31740819

[19] Restrepo, P. *et al*. Single-cell spatial transcriptomic analysis of human skin anatomy. *Nat Genet* **58**, 903–915 10.1038/s41588-026-02552-8 (2026). PMC13083264 41872488

[20] Franzen, O., Gan, L. M. & Bjorkegren, J. L. M. PanglaoDB: a web server for exploration of mouse and human single-cell RNA sequencing data. *Database (Oxford)* **2019**, 10.1093/database/baz046 (2019). PMC6450036 30951143

[21] Yu, G., Wang, L. G., Han, Y. & He, Q. Y. clusterProfiler: an R package for comparing biological themes among gene clusters. *OMICS* **16**, 284–287 10.1089/omi.2011.0118 (2012). PMC3339379 22455463

[22] Joost, S. *et al*. The Molecular Anatomy of Mouse Skin during Hair Growth and Rest. *Cell Stem Cell* **26**, 441–457 e447 10.1016/j.stem.2020.01.012 (2020). 32109378

[23] Liberzon, A. *et al*. The Molecular Signatures Database (MSigDB) hallmark gene set collection. *Cell Syst* **1**, 417–425 10.1016/j.cels.2015.12.004 (2015). PMC4707969 26771021

[24] Reynolds, G. *et al*. Developmental cell programs are co-opted in inflammatory skin disease. *Science* **371**, 10.1126/science.aba6500 (2021). PMC7611557 33479125

[25] Akula, S., Zhang, B., Riihimäki, M., Wernersson, S. & Raine, A. European Nucleotide Archive SRP690566 https://www.ebi.ac.uk/ena/browser/view/SRP690566 (2026).

[26] Akula, S., Zhang, B., Riihimäki, M., Wernersson, S. & Raine, A. 10.6084/m9.figshare.31980441 (2026).42816519

[27] Alcolea, M. P. & Jones, P. H. Lineage analysis of epidermal stem cells. *Cold Spring Harb Perspect Med* **4**, a015206 10.1101/cshperspect.a015206 (2014). PMC3869281 24384814

[28] He, H. *et al*. Single-cell transcriptome analysis of human skin identifies novel fibroblast subpopulation and enrichment of immune subsets in atopic dermatitis. *Journal of Allergy and Clinical Immunology* **145**, 1615–1628 10.1016/j.jaci.2020.01.042 (2020). 32035984

[29] Cohen, E. *et al*. Significance of stress keratin expression in normal and diseased epithelia. *iScience* **27**, 108805 10.1016/j.isci.2024.108805 (2024). PMC10828818 38299111

[30] van den Brink, S. C. *et al*. Single-cell sequencing reveals dissociation-induced gene expression in tissue subpopulations. *Nature Methods* **14**, 935–936 10.1038/nmeth.4437 (2017). 28960196

[31] O’Flanagan, C. H. *et al*. Dissociation of solid tumor tissues with cold active protease for single-cell RNA-seq minimizes conserved collagenase-associated stress responses. *Genome Biology* **20**, 210 10.1186/s13059-019-1830-0 (2019). PMC6796327 31623682

